# Successful rescue of a patient with cardiogenic shock following traumatic cardiac arrest using VA-ECMO after air medical transport: a case report

**DOI:** 10.3389/fcvm.2025.1552037

**Published:** 2025-02-04

**Authors:** Haohao Wu, Pin Lan, Kechun Zhou, Lutao Xie

**Affiliations:** Department of Emergency, The Fifth Affiliated Hospital of Wenzhou Medical University, Lishui Central Hospital, Zhejiang, China

**Keywords:** traumatic cardiac arrest, femoral artery and vein injury, hemorrhagic shock, cardiogenic shock, VA-ECMO, air medical transport, interhospital transfer, case report

## Abstract

**Background:**

Traumatic cardiac arrest (TCA) poses significant challenges in resuscitation, with extremely high mortality rates, making it a critical issue in emergency and critical care medicine. Veno-arterial extracorporeal membrane oxygenation (VA-ECMO) has emerged as a crucial rescue technology for patients with cardiac arrest, providing short-term support for cardiopulmonary failure. However, the successful application and related clinical experience of VA-ECMO in TCA remain limited and require further investigation.

**Case presentation:**

A male patient sustained a stab wound to the left lower limb, resulting in femoral artery and vein injuries, massive hemorrhage, and subsequent hemorrhagic shock. The patient experienced cardiac arrest upon admission to a local hospital. Following cardiopulmonary resuscitation (CPR) and emergency femoral vascular reconstruction surgery, spontaneous circulation was temporarily restored, but the patient remained hemodynamically unstable postoperatively. Initial treatment at the local hospital was ineffective. On the second morning, the patient was transferred to our hospital via air medical transport, with a transport time of 35 min. Upon arrival, the patient was promptly evaluated, and VA-ECMO support was initiated within 17 min. After 3 days of VA-ECMO support and 5 days of mechanical ventilation, the patient was successfully weaned from life support and discharged in good condition.

**Conclusion:**

VA-ECMO can significantly improve the survival outcomes of patients with cardiogenic shock following traumatic cardiac arrest. The use of interhospital air medical transport effectively reduces rescue time, providing critical opportunities for the timely management of severely ill patients.

## Background

TCA is associated with extremely high mortality and poor neurological outcomes. A study reported an overall in-hospital mortality rate of 73% for TCA, with a favorable neurological outcome rate of only 2% (1), primarily attributed to hemorrhagic shock (2). Addressing the underlying cause of hemorrhagic shock is crucial; in this case, emergency femoral artery and vein repair was performed to control bleeding from the vascular injury.

Due to the high fatality rate of TCA, cases of successful resuscitation followed by cardiogenic shock are rare. Cardiogenic shock after cardiac arrest is a life-threatening emergency. A retrospective study demonstrated that VA-ECMO support can improve survival outcomes in patients with cardiac arrest and cardiogenic shock (3). This report presents a case of a patient who sustained a stab wound causing left femoral artery and vein injury, resulting in massive hemorrhage, hemorrhagic shock, and subsequent cardiac arrest. Despite aggressive fluid resuscitation and blood transfusion at a local hospital postoperatively, the patient remained hemodynamically unstable, with suspected cardiogenic shock. The patient was transferred to our hospital via air medical transport and treated with VA-ECMO support, ultimately achieving full recovery and discharge.

## Case presentation

**Figure 1:**
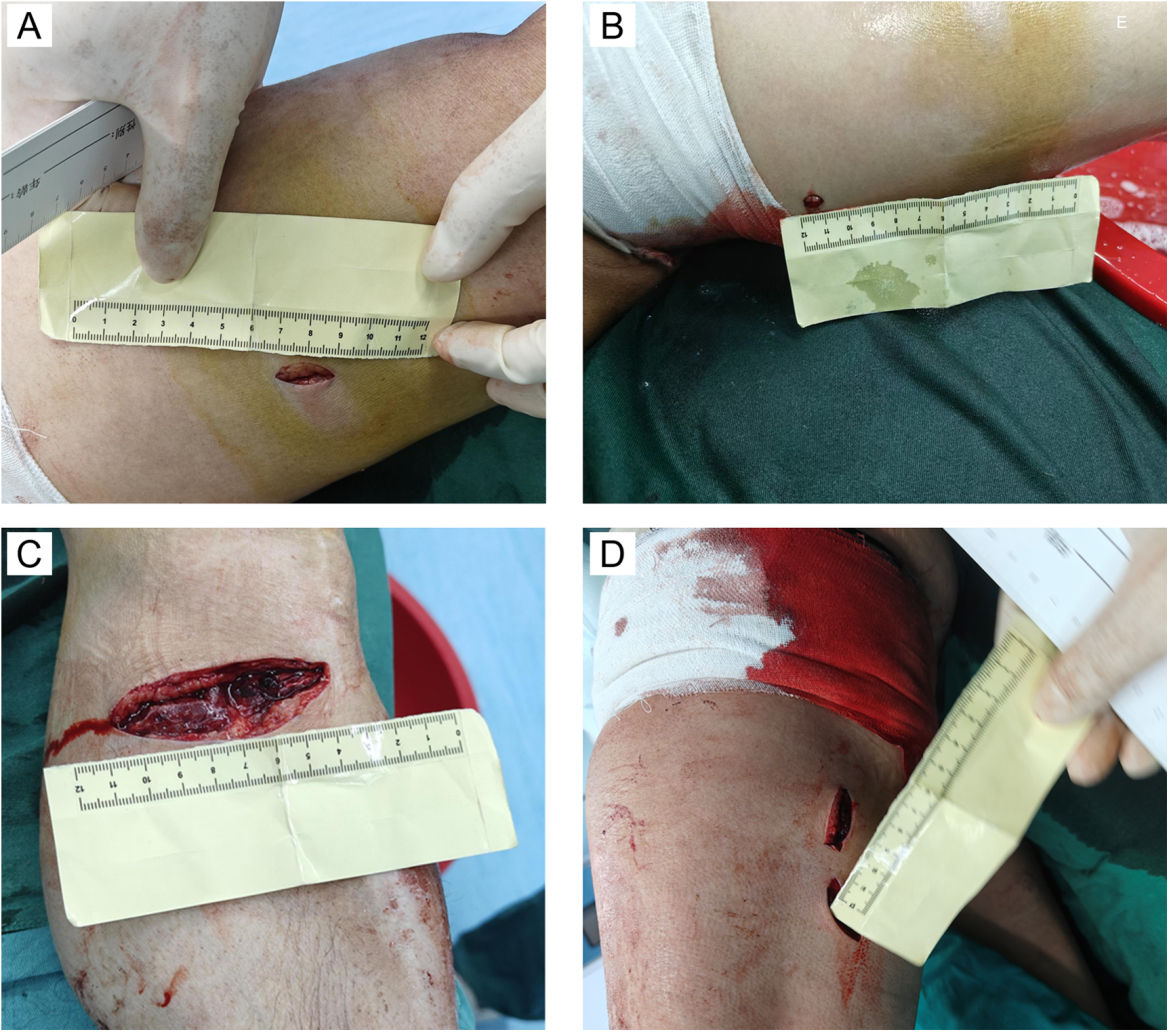
Stab wounds on the left lower extremity. **(A)** Wound on the lateral aspect of the left thigh; **(B)** Wound on the medial aspect of the left thigh; **(C)** Wound on the medial aspect of the left lower leg; **(D)** Wounds on the lateral aspect of the left lower leg.

**Figure 2:**
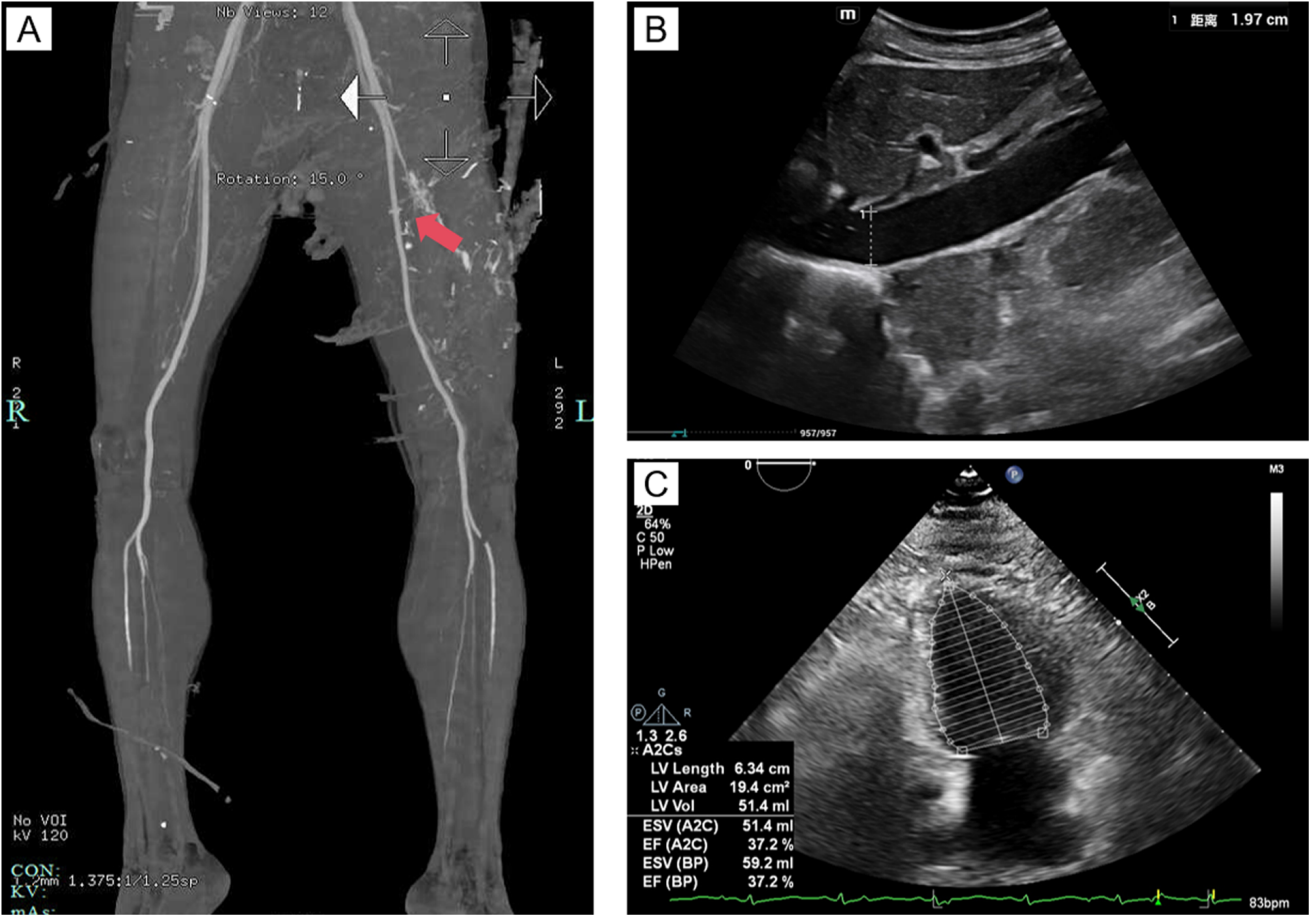
Imaging findings. **(A)** Contrast-enhanced CT of the lower extremities, with the red arrow indicating rupture and bleeding of the left femoral artery, with extravasation of contrast agent visible; **(B)** Ultrasound image of the inferior vena cava; **(C)** Echocardiographic image of the heart.

A 53-year-old male with no significant medical history was admitted to a local hospital emergency department one day before his transfer to our hospital after sustaining stab wounds to his left lower extremity. Upon arrival, his vital signs were as follows: body temperature 35°C, pulse 23 beats/min, blood pressure 44/18 mmHg, and oxygen saturation 69% on room air. The patient was unconscious, pale, and had cold extremities. Physical examination revealed multiple stab wounds on the left lower extremity: one on the lateral aspect of the left thigh (2 cm, Figure 1A), one on the medial aspect of the left thigh (1 cm, Figure 1B), one on the medial aspect of the left lower leg (7 cm, Figure 1C), and two on the lateral aspect of the left lower leg (2 cm each, Figure 1D). Initial management included hemostasis with a tourniquet and rapid transfusion and fluid resuscitation. However, the patient lost consciousness, and cardiac arrest was confirmed by continuous ECG monitoring. Blood pressure was unmeasurable. Advanced Cardiopulmonary Resuscitation (ACLS), including chest compressions, endotracheal intubation, mechanical ventilation, rapid fluid resuscitation, and blood transfusion, successfully restored spontaneous circulation (ROSC). After initial stabilization, contrast-enhanced CT of the lower extremities revealed a rupture of the left femoral artery and vein (Figure 2A).

A vascular and orthopedic surgical team from our hospital was dispatched to perform emergency left femoral artery and vein reconstruction and left gastrocnemius muscle tendon repair. Postoperatively, the patient was transferred to the ICU of the local hospital. Despite aggressive transfusion and fluid resuscitation, hemodynamic instability persisted the next morning, and cardiogenic shock was considered based on bedside echocardiography findings, which showed diffuse left ventricular hypokinesis. As the local hospital lacked ECMO facilities, an urgent air medical transfer to our hospital was arranged.

On the morning of the first day of admission, the patient was airlifted under the care of our medical transport team, comprising an attending physician and a nurse. The transport lasted only 35 min (a distance of 190 km), minimizing delays in initiating advanced treatment. Upon arrival at our hospital's emergency ICU, the patient remained hemodynamically unstable. Evaluation showed an inferior vena cava diameter of 1.97 cm (Figure 2B) and a left ventricular ejection fraction (EF) of 37.2% on echocardiography (Figure 2C), indicating post-cardiac arrest myocardial injury and cardiogenic shock. VA-ECMO was immediately initiated via femoral artery and vein cannulation on the contralateral side to the trauma to avoid complications at the injury site, along with mechanical ventilation and pharmacological support. Given the high risk of bleeding following vascular repair, anticoagulation was withheld during ECMO therapy. Coagulation function was closely monitored during ECMO support, and specific values are shown in Table 1.

**Table 1:** Daily record of hemodynamics, laboratory inspection, ventilatory parameters, and ECMO status over the first three days in EICU.

	^a^Day 1	Day 1	Day 2	Day 3
	Hour 0	Hour 6		
Hemodynamic parameters				
Heart rate (bpm)	86	91	63	79
Arterial pressure (mmHg)	79	141	108	121
Norepinephrine (mcg/kg/min)	1.78	1.07	0	0
Epinephrine (mcg/kg/min)	0.28	0	0	0
Laboratory inspection				
Hemoglobin (g/dl)	6.8	6.1	7.6	7.3
Hematocrit (%)	19.4	17.7	21.2	20.6
Platelets (per µl)	55,000	36,000	30,000	30,000
pH	7.25	7.447	7.46	7.46
pCO2 (mmHg)	26	30	31	37.4
pO2 (mmHg)	212	130	114	127
HCO3 (mmol/L)	11.7	15	21.1	26.8
BE (mmol/L)	−14.5	−8.0	−5.0	2.5
Lactate (mmol/L)	11.6	2.5	2.4	1.3
Myoglobin (ng/ml)	921.4	–	299.6	251.6
Troponin (ng/ml)	1.78	0.82	0.192	0.192
Serum creatinine (μmol/L)	140	96	64	69
APTT (s)	480	76.3	61.9	41.1
D-dimer (mg/L FEU)	1.85	4.61	16.67	19.63
Ventilatory parameters				
Respiratory rate	14	14	17	14
Oxygen saturation	–	100	97	98
Ventilation mode	VC	VC	PC	PC
FiO2 (%)	50	40	35	35
IMV (times/min)	14	14	14	14
PEEP (cm H2O)	10	10	8	5
Tidal volume (ml)	380	380	450	550
ECMO record				
Sweep gas flow (SGF) L/min	3.0	2.9	2.9	1.4
ECMO flow (L/min)	3.1	3.0	3.0	1.5

^a^ Day 1: refers to the second day after the injury, when admitted to EICU of our hospital.

During his EICU stay, the patient required VA-ECMO, norepinephrine, and epinephrine support, in addition to multiple blood transfusions. Laboratory tests showed gradual improvement in myocardial enzyme and lactate levels: troponin I decreased from 1.78 ng/ml on admission to 0.192 ng/ml before ECMO weaning, myoglobin decreased from 921.4 ng/ml to 251.6 ng/ml, and lactate levels significantly dropped from 11.6 mmol/L to 1.3 mmol/L, indicating improved tissue perfusion (Table 1). Hemodynamics stabilized under ECMO support, with a mean arterial pressure (MAP) consistently above 65 mmHg after discontinuing vasopressors and no malignant arrhythmias observed.

On the third day after admission, following comprehensive evaluation, ECMO was successfully weaned. Criteria for weaning included significant improvement in the underlying condition, echocardiographic evidence of a left ventricular EF of 50%, mechanical ventilation parameters with a tidal volume of 550 ml, a respiratory rate of 14 breaths/min, and an oxygen saturation of 98%. Hemodynamics remained stable (24-hour MAP >65 mmHg, pulse pressure >20 mmHg, no malignant arrhythmias), lactate levels were <2 mmol/L, and myocardial enzymes continued to decrease (Table 1).

**Figure 3:**
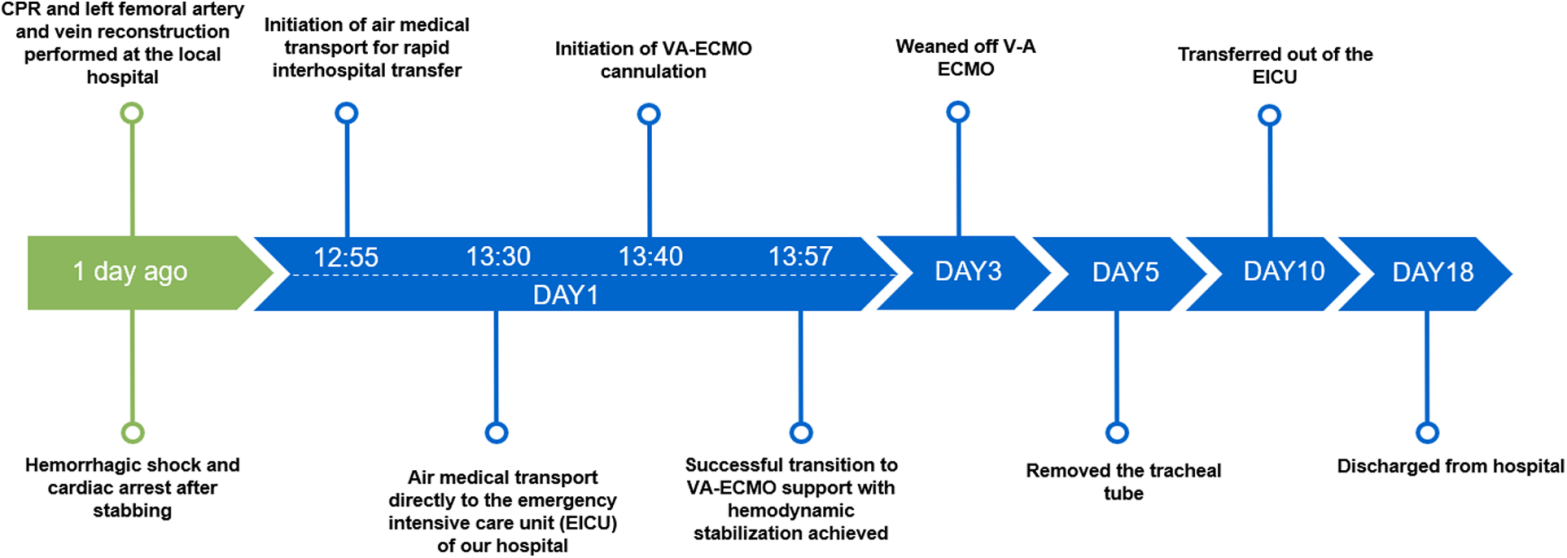
Patient's treatment course.

Following ECMO weaning, the patient remained intubated and required ventilator support, sedation, analgesia, infection control, and organ protection therapy. On the fifth day after admission, the endotracheal tube was successfully removed, and high-flow oxygen therapy was initiated. With continued supportive and rehabilitative care, the patient's condition improved, and he was transferred to the general ward on the tenth day after admission. He was discharged in stable condition after a total of 18 days of hospitalization. Follow-up was conducted via telephone, and the patient reported good physical function and mental well-being. The clinical course is summarized in Figure 3.

## Discussion

This case presents the successful rescue of a patient with cardiogenic shock following traumatic cardiac arrest through multidisciplinary intervention, aeromedical evacuation, and VA-ECMO support. By employing rapid transportation, a heparin-free anticoagulation strategy, and precise advanced life support, the patient ultimately achieved full recovery. This case highlights the advantages of integrated modern emergency care technologies and provides valuable insights for managing similarly complex trauma cases.

After the return of spontaneous circulation (ROSC) following traumatic cardiac arrest, the patient exhibited persistent haemodynamic instability requiring high doses of vasoactive agents, indicating myocardial injury. The mechanisms of myocardial injury after cardiac arrest include ischaemia-reperfusion injury and alterations in myocardial cell membrane permeability, leading to decreased myocardial contractility, reduced cardiac output, and inadequate tissue perfusion (4). Further investigations revealed a left ventricular EF of 37% on echocardiography and significantly elevated lactate levels, indicating worsening systemic hypoxia. Despite aggressive fluid resuscitation and blood transfusion, haemodynamic parameters showed no improvement, confirming a diagnosis of cardiogenic shock secondary to post-cardiac arrest myocardial injury. VA-ECMO has been increasingly employed in patients with cardiogenic shock. Multiple studies have demonstrated that VA-ECMO significantly improves survival in post-cardiac arrest cardiogenic shock by replacing cardiac and pulmonary function, maintaining haemodynamic stability, and enhancing tissue perfusion (5, 6), which provided critical support in the management of this case.

However, the use of VA-ECMO in patients with cardiogenic shock following traumatic cardiac arrest (TCA) remains relatively rare, primarily due to the high risk of bleeding in trauma patients. The selection of an appropriate anticoagulation strategy is critical when employing VA-ECMO in such cases. Recent advancements in coated circuit technology have demonstrated the safety and efficacy of heparin-free anticoagulation strategies in patients with a high risk of bleeding (7). Studies have shown that early heparin-free ECMO support can increase survival rates to as high as 60% in trauma patients with haemorrhagic shock, with some patients achieving complete recovery (8). In this case, early femoral arteriovenous reconstruction laid a crucial foundation for subsequent VA-ECMO support. Considering the patient's ongoing high bleeding risk, a heparin-free anticoagulation strategy was adopted during ECMO operation. Coagulation function and bleeding risk were dynamically assessed by monitoring fibrinogen levels, D-dimer, activated partial thromboplastin time (aPTT), and platelet count (9). This strategy successfully prevented further traumatic haemorrhagic complications, demonstrating that a heparin-free anticoagulation approach is both feasible and effective in TCA patients with cardiogenic shock. It provides valuable insights for the management of similar cases in the future.

Air Medical Transport (AMT) played a pivotal role in the management of this patient. Its primary advantage lies in the rapid transfer of patients to medical centres equipped with advanced life support capabilities, thereby reducing treatment delays and improving outcomes. Vuorinen P et al. (10)demonstrated that AMT significantly shortens transport times for critically ill patients in remote areas, providing a vital opportunity for timely intervention. This is particularly important in regions with mountainous terrain or uneven distribution of medical resources, where air rescue offers a crucial solution. Early identification and prompt treatment of cardiogenic shock are essential for improving patient prognosis (11). In this case, AMT facilitated the transfer of the patient over a distance of 190 kilometres within 35 min, securing a critical time window for advanced life support. The successful management of this patient further underscores the importance of AMT as an essential mode of rapid transport for trauma patients.

Although the patient in this case ultimately achieved recovery, several limitations remain. First, the nature of a case report inherently limits the generalisability of its findings, necessitating further studies to validate its applicability. Second, while the heparin-free anticoagulation strategy demonstrated favourable short-term outcomes, its long-term safety and the risk of thrombotic complications require additional clinical evidence. Furthermore, the air medical transport model highlighted disparities in the integration of medical resources. Future research should focus on optimising regional emergency networks, particularly enhancing the integration of air medical transport with advanced life support technologies, to further improve the overall management of trauma patients.

## Conclusion

This case demonstrates the successful application of multidisciplinary intervention, air medical transport, and VA-ECMO support in the management of a patient with cardiogenic shock following traumatic cardiac arrest. It also validates the safety and feasibility of a heparin-free anticoagulation strategy. This case provides valuable insights into the treatment of cardiogenic shock after TCA and highlights the critical role of promptly initiating advanced life support and air medical transport in maximising the time available for patient care.

## Data Availability

The original contributions presented in the study are included in the article/Supplementary Material, further inquiries can be directed to the corresponding author.
